# Social context as a source of variability in the psychological sciences

**DOI:** 10.3389/fnhum.2024.1507010

**Published:** 2025-01-09

**Authors:** Laura A. Agee, Abdellah Fourtassi, Marie-H. Monfils

**Affiliations:** ^1^Department of Neuroscience, The University of Texas at Austin, Austin, TX, United States; ^2^Aix Marseille Université, CNRS, LIS, Marseille, France; ^3^Department of Psychology, The University of Texas at Austin, Austin, TX, United States

**Keywords:** social context, emotional contagion, reproducibility of results, dominance—rank orders, social instability

## Introduction

Poor reproducibility in the psychological sciences is often attributed to systemic factors such as publication bias and lack of financial support sources for replication studies (Open Science Collaboration, 2015). While such factors undoubtedly contribute to the problem, controllable issues such as variability in testing methodology, laboratory environment, and subject characteristics may serve as other possible sources of non-replication (Van Bavel et al., 2016; Crabbe et al., 1999; Sorge et al., 2014). One source of variability which is rarely accounted for in either human or animal studies is social context, i.e., the environment formed as a result of the behavioral and biological characteristics of the conspecifics with whom the subject interacts or coexists. Alongside more direct social influences (e.g., conspecific aggression), social context may also be influenced by broader, indirect influences arising from trends in conspecific behavior/beliefs (e.g., cultural norms) or the subject's place in the broader social order (e.g., dominance status). Here, we will cover some of the ways in which preventable variations in social context might influence behavioral measures in human and non-human animal research. We will then discuss strategies to account for social context in future research.

## Effects of social context on behavior in non-human animals

**Figure 1:**
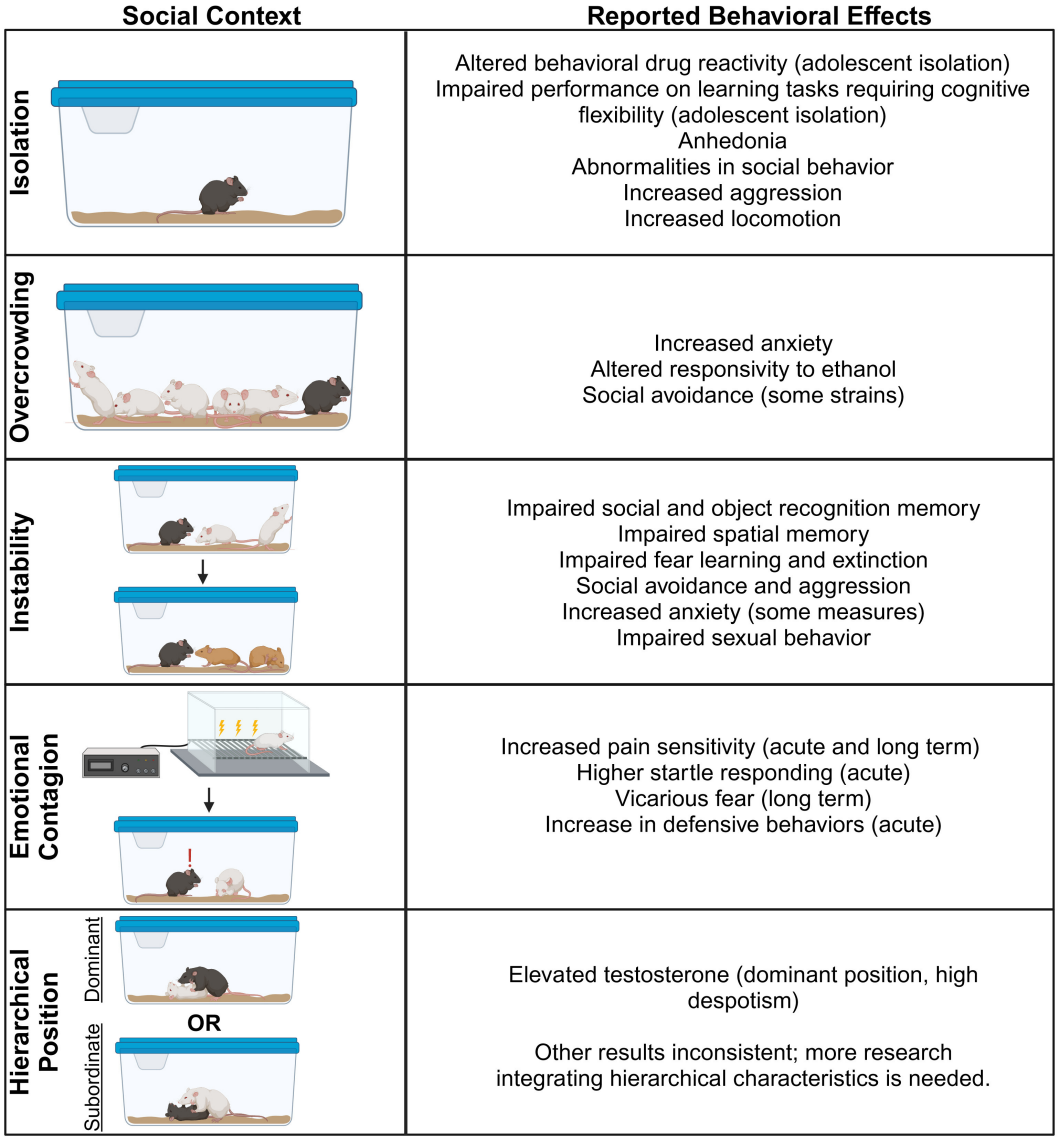
Social contextual factors and some of their influences on behavior in non-human animals.

(See Figure 1 for section overview).

First, we consider the overall population density of the home cage. It is well-established that in social species such as mice, rats, and non-human primates, extended periods of social isolation produce a range of marked behavioral abnormalities (Valzelli, 1973; McKinney, 1974; Love and Zelikowsky, 2020). Social isolation has been found to impair various forms of learning (Einon, 1980; Lander et al., 2017), induce abnormal social behaviors (McKinney, 1974; Koike et al., 2009; Mitchell et al., 1966; Keesom et al., 2017; Rivera-Irizarry et al., 2020), increase locomotion (Lander et al., 2017; Ieraci et al., 2016), alter behavioral drug responsivity (Lander et al., 2017; Wongwitdecha and Marsden, 1996), increase aggressive behavior toward conspecifics (Koike et al., 2009; Mitchell et al., 1966; Wongwitdecha and Marsden, 1996), and exacerbate behavioral markers of anxiety and depression (Lander et al., 2017; Koike et al., 2009; Ieraci et al., 2016; Weiss et al., 2004; Lukkes et al., 2009). These effects vary depending on species, sex, and the age at social isolation. On the opposite end of the spectrum, overcrowding may also serve as a source of stress and behavioral abnormalities. In mice, high population density (< 8–15 in^2^ surface area in the cage/mouse) [National Research Council (US) Committee for the Update of the Guide for the Care and Use of Laboratory Animals, 2011] has been found to increase adiposity, produce an anxiety-like phenotype, alter behavioral responsivity to ethanol and ethanol sensitization, and—in some strains—induce social avoidance (Lin et al., 2015; Delaroque et al., 2021; Lee et al., 2018; van Ingelgom et al., 2024; Laber et al., 2008).

An unstable social context can also serve as a source of stress resulting in behavioral changes. For example, mice subjected to chronic social instability (CSI) stress by having cage mates repeatedly replaced with novel conspecifics over the course of multiple weeks display impaired recognition memory (Featherstone et al., 2022), spatial memory (Schmidt et al., 2010), and social memory (Saavedra-Rodríguez and Feig, 2013). Additionally, CSI subjugated mice display social avoidance (Saavedra-Rodríguez and Feig, 2013; dos Santos Guilherme et al., 2022), increased social aggression (Schmidt et al., 2007), and behavioral patterns consistent with anhedonia (Featherstone et al., 2022; Schmidt et al., 2010; Dadomo et al., 2018; Haller et al., 1999; Koert et al., 2021; de Lima and Massoco, 2017) and increased anxiety (Schmidt et al., 2010; Saavedra-Rodríguez and Feig, 2013; dos Santos Guilherme et al., 2022; Koert et al., 2021; Yohn et al., 2019) (excepting in the open field test, see Featherstone et al., 2022; Dadomo et al., 2018; de Lima and Massoco, 2017; Sturman et al., 2021; Díez-Solinska et al., 2022). In rats, CSI produces long-term spatial and social/object recognition memory deficits (Green and McCormick, 2013; McCormick et al., 2010, 2012; Hodges et al., 2017), impairs fear learning (Morrissey et al., 2011) and extinction (McCormick et al., 2013b), reduces social approach (Hodges et al., 2017; Green et al., 2013; Graf et al., 2023; Hodges et al., 2018), impairs sexual behavior (McCormick et al., 2013a), and increases defensive social behavior (Graf et al., 2023). As with social isolation, behavioral effects of CSI vary based on species, sex, and the timing of the procedure/behavioral testing (see Koert et al., 2021 for review). Notably, while the vast majority of experiments involve multiple weeks of CSI, some of the behavioral changes could be observed as early as 2 days into the CSI procedure (Dadomo et al., 2018). Furthermore, mice moved to a new social context after an hour of isolation display higher levels of corticosterone compared to controls (McCormick et al., 2007; Hodges et al., 2014). This suggests that some of the behavioral effects observed following CSI could manifest acutely after even minor shuffling of research subjects.

The individual experiences of group members also influence behavior in their cage mates via emotional contagion, i.e., the psychological phenomenon whereby observing a change in another individual's behavior activates this same change in behavior in the viewer (Panksepp and Lahvis, 2011). In rodents, it is well established that directly observing a conspecific in distress causes acute physiological and behavioral changes, such as increases in fear-related/defensive behavior (Jeon et al., 2010; Bruchey et al., 2010; Andraka et al., 2021; Keysers and Gazzola, 2021), enhanced startle responding, and hyperalgesia (Langford, 2006; Li et al., 2014). Moreover, observing a conspecific in distress can induce extended effects such as long-term pain sensitization (Raber and Devor, 2002) and fear responses to stimuli that accompanied conspecific distress (Jeon et al., 2010; Bruchey et al., 2010; Kavaliers et al., 2001). Observing pain or distress in familiar or related conspecifics often produces more potent behavioral effects and is sometimes necessary for long-term effects to be observed in both mice and rats (Jeon et al., 2010; Langford, 2006; Li et al., 2014; Agee et al., 2019; Jones et al., 2014; Kavaliers et al., 2005) (though see Hernandez-Lallement et al., 2022). This suggests a keen sensitivity toward the emotional state of cage mates. In this way, the treatment of a given subject may be sufficient to alter their cage mates' behavior either acutely (e.g., if animals within a single cage are run sequentially and allowed to interact between testing) or over the long term (e.g., if a cage mate is subjected to surgical or testing procedures that cause enduring stress/pain).

Finally, we consider the influence of social rank on behavior. Most socially housed laboratory species are known to maintain dominance hierarchies to some degree (Williamson et al., 2016, 2019; Varholick et al., 2019; Schuhr, 1987; Blanchard et al., 1984; Ziporyn and McClintock, 1991; Sterck and Steenbeek, 1997; Blanchard et al., 1988; Jones and Monfils, 2016; Seese et al., 2024; Monfils and Agee, 2019), but to simplify our discussion we will focus on mice. The results of the dominance literature regarding the effect of social rank on behavior are quite inconsistent. A recent meta-analysis (Varholick et al., 2021) found no clear effect of dominance rank across studies in open field exploration, elevated plus maze open arm time, or immobility during the forced swim test. Indeed, results were often directly contradictory. One explanation for this lack of consensus is variability in the type of dominance hierarchy formed within a group. In triads of mice, variation is observed both in the stability of a hierarchy (i.e., the degree to which rank is maintained) and the linearity of the hierarchical structure (Varholick et al., 2019). Reports of overall hierarchical stability vary between studies, with some researchers finding high stability (Williamson et al., 2016, 2019) and others reporting frequent reshuffling of rank order (Varholick et al., 2019). The degree of alpha despotism, i.e., the ability to suppress aggressive behavior in lower ranked counterparts, also varies (Williamson et al., 2016). This variance is important to consider, as recent research has found that the often-inconsistent findings regarding endocrine function and behavior in dominant vs. subordinate animals may be explained by interactions between dominance rank and hierarchical characteristics. For example, while past research has found contradictory results on the relative testosterone levels in dominant and subordinate mice (Machida et al., 1981; Ely, 1981; Selmanoff et al., 1977; Barnard et al., 1996; Hilakivi et al., 1989), recent evidence suggests that high despotism may serve as the determining factor for this difference (Williamson et al., 2017). Further research considering hierarchical characteristics in conjunction with social rank will hopefully resolve some of these contradictions.

## Effects of social context on participant behavior in human studies

The social context of human subjects will virtually always be more complex than that of lab animals confined to a fixed community of only a few conspecifics. As such, human researchers can realistically only hope to assess participants based on broad differences in social context in which individuals can be easily categorized or scored. We thus restrict this discussion to a few facets of an individual's social context that can reasonably be ascertained from basic participant surveys. Additionally, we discuss how more immediate aspects of the social context during testing (e.g., the presence of an experimenter or other subjects) might affect responding.

As in many lab species, social isolation in human subjects has been shown to be associated with a variety of physiological and behavioral effects. For example, individuals reporting high subjective social isolation display higher levels of depression (Fiordelli et al., 2020; Steptoe et al., 2013; Layden et al., 2017), increased mortality (Steptoe et al., 2013; Holt-Lunstad et al., 2015), and generally interpret social interactions more negatively (Duck et al., 1994; Anderson and Martin, 1995; Hawkley et al., 2003). Notably, in humans, perceived social isolation (i.e., loneliness) is measurable and distinct from objective social isolation (i.e., actual social network size), and the two measures correlate only weakly to moderately (Fiordelli et al., 2020; Steptoe et al., 2013; Hawkley et al., 2008) (see also Layden et al., 2017). Additionally, the quality of social ties—not the number of ties—appears to exert a greater protective influence on loneliness levels (Lee and Ko, 2018). As such, simply gathering demographic data may not be an accurate gauge of social context.

The immediate social environment during testing also has the potential to alter participant responding. When studying social behavior, lab studies have traditionally used non-participatory settings, where people observe stimuli of others without being part of the interaction. While this research is valuable in documenting human social biases in general, it fails to account for people's true social behavior outside the lab (Risko et al., 2016; Pfeiffer et al., 2012). For example, in the case of social attention, non-participatory settings tend to overemphasize face gazing as an information-gathering tool; however, whether we know that our gaze is available to others has significant consequences on how much we look at them (Laidlaw et al., 2011; Gobel et al., 2015). More specifically, non-participatory lab experiments overlook the effect of gaze as a signaling tool in natural social interactions (Risko et al., 2016). Crucially, this effect is modulated by cultural norms, relationships between interactants (e.g., familiar person vs. stranger), and the nature of their interaction (e.g., cooperative vs. collaborative), emphasizing the necessity to factor socio-contextual features in studies (Dalmaso et al., 2020).

This observation has broad implications beyond the study of social behavior. Human research generally involves interactions with a human experimenter in some way or another. This is particularly the case in child development research, since young children cannot, for example, read instructions off a computer screen. This makes the experimental outcome partly dependent on the experimenter-child social dynamics. Take, for example, the so-called Marshmallow task (Mischel et al., 1972) introduced to test children's delayed gratification management. Kidd et al. (2013) showed that children's perception of the experimenter's trustworthiness influences their strategy. In fact, the experimenter's identity alone (e.g., perceived as in-group vs. outgroup) had a significant impact on the wait time in the task (Pierre et al., 2023; Strickland, 1972). Furthermore, children's performance depends on their cultural background. For instance, societies that emphasize hierarchy vs. autonomy lead the child to adopt different self-regulatory strategies (Lamm et al., 2018). Similarly, children tend to adopt strategies that are consistent with their cultural norms related to waiting and food (Yanaoka et al., 2022). Failing to consider the social context (or lack thereof) can impact both the external validity of behavioral tasks as well as their internal validity, potentially contributing to the replicability crisis in human research.

## Discussion

In the preceding sections we highlighted some of the ways in which social context influences behavioral and physiological measures. While we do not have space to cover all components of social context here, what we have reviewed hopefully makes a compelling case for the idea that even experiments not focused on social behavior should be designed with certain aspects of social context in mind. Controlling all aspects of social context is not feasible, but some basic measures can be taken to limit social confounds. In non-human animal studies, social context can be standardized across experimental groups and between studies (if replication is the goal) by careful housing practices aimed at minimizing social stressors. In practice, however, this is rarely straightforward. For example, emotional contagion can theoretically be minimized by keeping subjects in single housing, but this exposes subjects to the behavioral and physiological changes that accompany social isolation stress. In such cases, alternative solutions—e.g., ensuring a balanced distribution of members of each experimental group between cages—should also be considered. Critically, details on social housing conditions and experimental group distribution between cages should be explicitly stated in the methods section and recorded on publicly available datasheets. Having this information readily available will help in interpreting inconsistent results and could assist researchers conducting meta-analyses.

Naturally, controlling for social context in human research is a more complicated prospect. While experimenters have no control over the broader aspects of their human participant's social context, variability in social context can be at least considered in analyses. Basic details of participants' social relationships might be gleamed via pre-screening or post-testing questionnaires and assessed as possible response mediators, but more detailed questions about the quality of these relationships may be necessary to properly categorize participants. Additionally, careful consideration must be given to the nature of interactions between participants and other individuals present during testing. As with animal research, thorough documentation of these interactions is essential for later interpretation of inconsistent results. While accounting for social context in research presents a formidable challenge, it is essential to consider for improving reproducibility and the validity of behavioral studies. By prioritizing the standardization and documentation of social variables, researchers can mitigate potential confounds and contribute to a more reliable body of scientific knowledge.
